# Weight-loss-independent benefits of exercise on liver steatosis and stiffness in Japanese men with NAFLD

**DOI:** 10.1016/j.jhepr.2021.100253

**Published:** 2021-02-10

**Authors:** Sechang Oh, Takehiko Tsujimoto, Bokun Kim, Fumihiko Uchida, Hideo Suzuki, Seiichiro Iizumi, Tomonori Isobe, Takeji Sakae, Kiyoji Tanaka, Junichi Shoda

**Affiliations:** 1 Faculty of Medicine, University of Tsukuba, Tsukuba, Ibaraki, Japan; 2 Faculty of Human Sciences, Shimane University, Shimane, Japan; 3 Department of Sports Health Care, Inje University, Gimhae, Republic of Korea; 4 Department of Oral and Maxillofacial Surgery, University of Tsukuba Hospital, Tsukuba, Ibaraki, Japan; 5 Doctoral Program in Clinical Sciences, Graduate School of Comprehensive Human Sciences, University of Tsukuba, Tsukuba, Ibaraki, Japan; 6 Faculty of Health and Sport Sciences, University of Tsukuba, Tsukuba, Ibaraki, Japan

**Keywords:** Aerobic exercise, Dietary restriction, Liver fat, Liver stiffness, Hepatokine, Myokine, Nuclear factor-erythroid 2-related factor 2, ALT, alanine aminotransferase, ANGPTL6, angiopoietin-like 6, AST, aspartate aminotransferase, BDNF, brain-derived neurotrophic factor, CAP, controlled attenuation parameter, E_large_, large amount of exercise group, E_small_, small amount of exercise group, E_sub_, exercise (subset for which biological samples were available) group, E_total_, exercise group, FGF-21, fibroblast growth factor-21, FAST-Score, FibroScan-AST score, FPG, fasting plasma glucose, GGT, gamma-glutamyl transpeptidase, GCLC, glutamate-cysteine ligase catalytic subunit, GCLM, glutamate-cysteine ligase modifier subunit, GPx, glutathione peroxidase, HO1, heme oxygenase 1, HOMA-IR, homeostasis model assessment-insulin resistance, KC, Kupffer cells, LPS, lipopolysaccharide, LSM, liver stiffness measured using transient elastography, mnSOD, manganese superoxide dismutase, MVPA, moderate-to-vigorous intensity physical activity, NAFLD, non-alcoholic fatty liver disease, NASH, non-alcoholic steatohepatitis, NEFAs, non-esterified fatty acids, NF-Score, NAFLD fibrosis score, NQO1, NAD(P)H quinone oxidoreductase, Nrf2, nuclear factor E2-related factor 2, PBMCs, peripheral blood mononuclear cells, Se-P, selenoprotein-P, SPARC, secreted protein acidic and rich in cysteine, TBARS, thiobarbituric acid-reactive substances, TEI, total energy intake, TG, triglycerides, TNF-α, tumour necrosis factor alpha, VAT, visceral adipose tissue, WC, waist circumference, WFA^+^-M2BP, *Wisteria floribunda* agglutinin-positive human Mac-2 binding protein, W_sub_, weight-loss (subset for which biological samples were available) group, W_total_, weight-loss group

## Abstract

**Background & Aims:**

A weight-loss-independent beneficial effect of exercise on non-alcoholic fatty liver disease (NAFLD) management has been reported, but the underlying mechanism is unknown. To help determine this mechanism, the effects of exercise on individual tissues (liver, adipose tissue, and skeletal muscle) were retrospectively studied.

**Methods:**

Data from Japanese obese men with NAFLD in a 3-month exercise regimen were analysed and compared with those in a 3-month dietary restriction program designed to achieve weight loss. The underlying mechanism was studied in a smaller subcohort.

**Results:**

Independent of the effect of weight loss, the exercise regimen reduced liver steatosis by 9.5% and liver stiffness by 6.8% per 1% weight loss, and resulted in a 16.4% reduction in FibroScan-AST score. Improvements in these hepatic parameters were closely associated with anthropometric changes (reduction in adipose tissue and preservation of muscle mass), increases in muscle strength (+11.6%), reductions in inflammation and oxidative stress (ferritin: -22.3% and thiobarbituric acid: -12.3%), and changes in organokine concentrations (selenoprotein-P: -11.2%, follistatin: +17.1%, adiponectin: +8.9%, and myostatin: -21.6%) during the exercise regimen. Moreover, the expression of target genes of the transcription factor Nrf2, an oxidative stress sensor, was higher in monocytes, suggesting that Nrf2 is activated. Large amounts of high-intensity exercise were effective at further reducing liver steatosis and potentiating improvements in pathophysiological parameters (liver enzyme activities and organokine profiles).

**Conclusions:**

The weight-loss-independent benefits of exercise include anti-steatotic and anti-stiffness effects in the livers of patients with NAFLD. These benefits seem to be acquired through the modification of inter-organ crosstalk, which is characterised by improvements in organokine imbalance and reductions in inflammation and oxidative stress.

**Lay summary:**

We investigated the effects of exercise on non-alcoholic fatty liver disease (NAFLD) that were not related to weight loss. We found that exercise had considerable weight-loss-independent benefits for the liver through a number of mechanisms. This suggests that exercise is important for NAFLD patients, regardless of whether they lose weight.

## Highlights

• Exercise has effects on liver steatosis and stiffness, independent of weight loss.
• Exercise maintains muscle mass and alters the secretion of organokines.
• Exercise increases the phagocytic capacity of Kupffer cells and activates Nrf2.
• Exercise, especially vigorous exercise, should be used aggressively to manage non-alcoholic fatty liver disease (NAFLD).

## Graphical abstract

**Figure:**
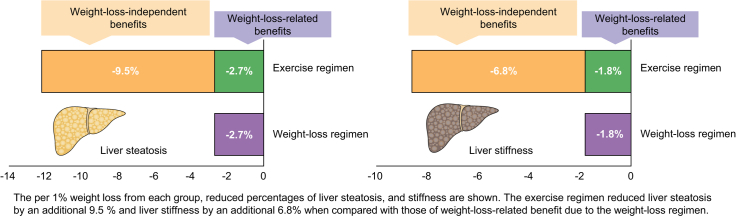

## Introduction

Non-alcoholic fatty liver disease (NAFLD) is regarded as a public health concern because of its high prevalence and association with the onset and progression of hepatic dysfunction: Of those with NAFLD, 25% will progress to non-alcoholic steatohepatitis (NASH).^1^ In addition, NAFLD increases the risks of metabolic, neoplastic, cardiovascular events; and more generally, of obesity-related morbidity and mortality.^2^ In Japan, cross-sectional data of the general population from 2009 to 2010 showed that the prevalence of NAFLD in middle-aged men was 41.0%, which indicates an especially high risk for men of this age group.^3^

Weight loss, as the result of lifestyle modifications, including dietary and exercise therapies, remains the cornerstone of NAFLD management.^4^ In particular, a level of dietary restriction has been recommended that aims to achieve the maximum calorie deficit in a short period of time. With regard to the weight loss pyramid for NAFLD, a previous study has shown that losses of at least ≥5% and ≥10% of body weight are necessary to reduce fat accumulation and fibrosis, respectively, in the liver.^5^

However, achieving and/or maintaining weight loss in the absence of a well-designed approach and expert support is difficult, therefore many patients with NAFLD fail in their attempts to lose weight. Moreover, most patients who successfully lose weight regain it over the following few months to years.^6^ Therefore, an additional therapeutic option beyond simple weight loss should be considered for effective NAFLD management.^7^

The use of exercise alone was not recommended as a therapy for NAFLD until quite recently. Previously, it had been just an add-on to a dietary approach to achieve a calorie deficit. However, the weight-loss-independent effects of exercise have recently received a great deal of attention.^8^ Because the optimal exercise prescription for patients with NAFLD has yet to be established, a number of studies have recently investigated the type and amount of exercise that would be most appropriate. As a result, significant improvements in NAFLD have been achieved following exercise interventions, in the absence of significant weight loss.^8^^,^^9^ A previous cohort study revealed that the stage of liver fibrosis in NAFLD is an independent predictor of mortality and liver-related events.^1^ Therefore, we wondered whether the weight-loss-independent benefits of regular exercise include an anti-stiffness effect. If so, an exercise regimen should be diligently followed to assist with hepatic rehabilitation by preventing liver fibrosis. However, the mechanisms underlying these potential benefits remain to be determined.

In this study, we aimed to identify these mechanisms using a retrospective study, with special reference to the potential anti-steatotic and anti-stiffness effects of an exercise regimen and its effects on individual organs (liver, adipose tissue, and skeletal muscle). The results of an exercise regimen were compared with those of a standard dietary weight-loss regimen to identify any advantage of regular exercise in the management of NAFLD.

## Materials and methods

### Participants

**Fig. 1:**
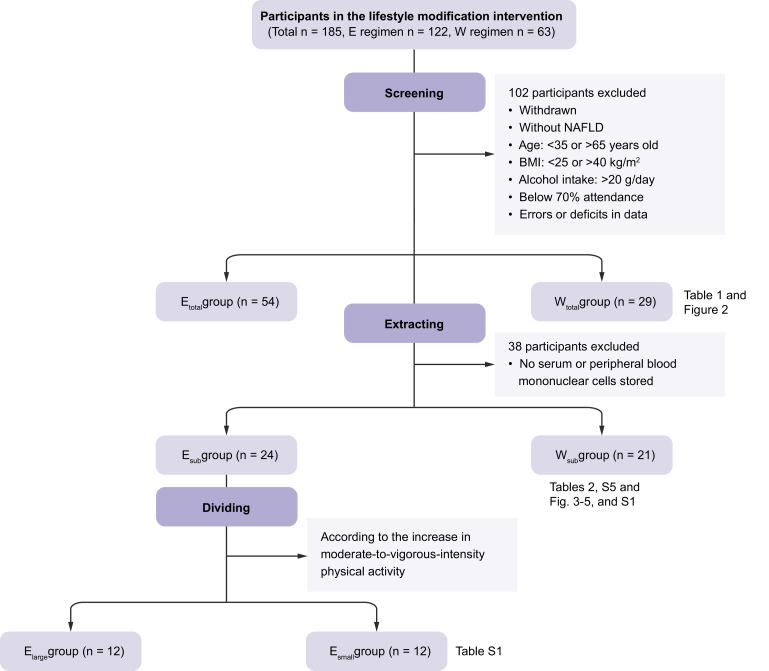
Flow diagram of the enrolment and classification of the study participants. E_large_, large amount of exercise, E_small_, small amount of exercise; E_sub_, exercise (subset for which biological samples were available); E_total_, exercise group; W_sub_ weight-loss (subset for which biological samples were available); W_total_, weight-loss group.

Figure 1 shows the workflow for the acquisition of data, which were collected between 2012 and 2015 at the University of Tsukuba, Ibaraki, Japan. Over this period, 185 men with obesity (Japanese criteria: BMI ≥25 kg/m^2^) who were living in Ibaraki Prefecture undertook, depending on their preference, either aerobic exercise or dietary restriction to induce weight loss over a 3-month period.

We excluded patients who withdrew from the programs who did not have NAFLD,^10^ who were <35 or >65 years old, those with a BMI >40 kg/m^2^, those with an alcohol intake of >20 g/day, those whose attendance rate was <70%, and those for whom there were errors or deficits in the data. Therefore, of the original 185 patients, data relating to 83 (exercise group, E_total_: n = 54; weight-loss group, W_total_: n = 29) were analysed.

For each of these patients, we checked whether stored serum, plasma, and peripheral blood mononuclear cells (PBMCs) were available, and used these samples to make further measurements in subgroups of the participants (E_sub_: n = 24 and W_sub_: n = 21, respectively). This permitted us to analyse additional parameters that might mediate the weight-loss-independent effects of exercise, such as potential risk factors for the progression of hepatic fibrosis, organokines, and the expression of nuclear factor erythroid 2-related factor 2 (Nrf2) target genes.

Furthermore, the E_sub_ group was divided into 2 subgroups: a group that undertook a large amount of exercise (E_large_) and a group that undertook a small amount of exercise (E_small_). The details are shown in Table S1.

These interventions were carried out in accordance with the principles of the Declaration of Helsinki and the study protocol was approved by the ethics committee of the Institutional Review Board at the University of Tsukuba (25-123, 6 July 2013 and 27-82, 6 January 2015). All the participants provided their written informed consent before enrolling in the study.

### Exercise regimen

Participants in the exercise groups took part in an aerobic exercise program. This program consisted of sessions of 90 min, 3 days a week for 3 months, and was supervised by professional trainers. The participants performed aerobic exercise of progressively increasing intensity, comprising fast walking and/or light jogging, and the most appropriate amount and intensity of exercise was recommended for each participant according to their daily condition and fitness goals by the trainers in an initial training session. At this time, the trainers asked the participants to perform as much exercise as possible, including on days when they were not under the trainers’ direct supervision.

### Weight-loss regimen

Participants in the weight-loss groups restricted their daily caloric intake to approximately 1,680 kcal/day. This program involved sessions of 90 min, once a week for 3 months, which were conducted by registered dietitians. We asked each participant to maintain their daily physical activity for 3 months. The detailed methodology of the program has been described previously.^9^

### Total energy intake

For daily assessment of the energy intake by participants, they completed a 3-day food record at baseline and after 3 months. Dietitians then calculated the total energy intake (TEI) and macronutrient composition of the diets using Eiyo-kunn version 4 (Kepakusya, Tokyo, Japan).

### Body composition

The fat mass and lean mass of each participant were measured by dual-energy X-ray absorptiometry using a QDR 4500 (Hologic, Bedford, MA, USA). Waist circumference (WC) was measured using a fiberglass tape at the level of the umbilicus.^11^

### Muscle strength

The methods used are described in Figure S1.

### Liver stiffness, steatosis, and Kupffer cells phagocytosis

A clinical gastroenterologist evaluated liver stiffness using transient elastography and steatosis using the controlled attenuation parameter, measured with a FibroScan 502 (Echosens, Paris, France). The Kupffer cell (KC) phagocytosis was assessed using contrast-enhanced ultrasonography. The principle and methodology of this technique have been described elsewhere.^9^^,^^12^

### Blood biochemistry

The methods used to measure aspartate aminotransferase (AST), alanine aminotransferase (ALT), and gamma-glutamyl transpeptidase (GGT) activity; and the insulin, glucose, ferritin, triglyceride (TG), and non-esterified fatty acids (NEFAs) are shown in Table S2. The commercial kits used to perform ELISA or electrochemiluminescence assays of thiobarbituric acid-reactive substances (TBARS), IL-6, leptin, fibroblast growth factor-21 (FGF-21), fetuin-A, secreted protein acidic and rich in cysteine (SPARC), brain-derived neurotrophic factor (BDNF), follistatin, M30, adiponectin, selenoprotein-P (Se-P), myostatin, angiopoietin-like 6 (ANGPTL6), decorin, *Wisteria floribunda* agglutinin-positive human Mac-2 binding protein (WFA^+^-M2BP), and fetuin-A are listed in Table S3.

### Surrogate markers

We evaluated insulin resistance using the homeostasis model assessment-insulin resistance (HOMA-IR)^13^ and calculated the NAFLD fibrosis score (NF-Score),^14^ FIB-4 index,^15^ and FibroScan-AST score (FAST-Score).^16^

### Real-time quantitative PCR

The methods used for PBMCs isolation and real-time quantitative PCR have been described in detail previously.^9^ The primers used are shown in Table S4.

### Statistical analysis

Statistical analysis was conducted using SPSS version 25.0 (IBM, Armonk, NY, USA). The results are presented as means (SEMs) of raw data, or log-transformed data if their distributions were skewed. To characterise intra-group changes over time, dependent variables were analysed using the paired *t* test. Differences between groups were identified using the independent *t* test. Finally, when data were adjusted for baseline values, the analysis of covariance (ANCOVA) was conducted. A value of *p* < 0.05 was considered to represent statistical significance.

## Results

### Baseline analysis

The age, attendance rate, and anthropometric characteristics did not differ significantly between the groups. However, there were significant differences in the TEI (*p* = 0.031), protein intake (*p* = 0.013), and NEFA (*p* = 0.025) between the E_total_ and W_total_ groups, and in KC phagocytosis (*p* = 0.026), leptin (*p* = 0.036), BDNF (*p* = 0.015), _log_*GPX* (*p* = 0.020), and _log_*mnSOD* (*p* = 0.029) between the E_sub_ and W_sub_ groups. These data were analysed with adjustments for the baseline values for each group.

### Equivalence in both parent-groups (n = 83) and subgroups (n = 45)

No statistically significant differences were found for all parameters (anthropometric characteristics, liver enzyme activities, surrogate markers of NASH and fibrosis, insulin resistance, lipid profile, and daily dietary intake) except for the FIB-4 index and fasting plasma glucose in the parent-groups (Table 1) and subgroups (Table 2). Also, the direction of the increase and decrease of the FIB-4 index and FPG in the exercise and weight-loss groups after 3 months was identical (Table 1, Table 2). Therefore, we judged that the parent-groups and subgroups were similar. Based on this background, we proceeded with the evaluation of the subgroups (Table 2).

**Table 1:** Outcomes in 83 obese men with NAFLD who participated in an exercise regimen (E_total_) or weight-loss regimen (W_total_).

	E_total_ (E_T_)			W_total_ (W_T_)			ΔE_T_*vs*. ΔW_T_
Parameters	Before	After	Δ	Before	After	Δ	*P*
n	54			29			
Age, years	49.8 (0.9)			52.4 (1.6)			0.127
Attendance, %	85.4 (1.0)			87.9 (1.6)			0.174
Anthropometric characteristics							
Body weight, kg	83.1 (1.4)	82.1 (1.4)	-1.0	82.4 (1.8)	74.1 (1.7)	-8.3^†^	E_T_ < W_T_^†^
BMI, kg/m^2^	28.2 (0.4)	27.9 (0.5)	-0.3∗	29.0 (0.5)	26.1 (0.4)	-2.9^†^	E_T_ < W_T_^†^
WC, cm	97.8 (1.0)	96.1 (1.1)	-1.6∗	99.4 (1.4)	90.1 (1.4)	-9.3^†^	E_T_ < W_T_^†^
Fat mass, kg	21.3 (0.7)	20.2 (0.7)	-1.1^†^	21.5 (0.9)	16.3 (0.8)	-5.2^†^	E_T_ < W_T_^†^
Lean mass, kg	62.3 (0.9)	62.4 (0.9)	+0.1	61.3 (1.4)	57.4 (1.3)	-3.9^†^	E_T_ > W_T_^†^
Liver enzyme activities							
AST, U/L	27.5 (2.1)	25.3 (1.5)	-2.2∗	25.5 (1.4)	21.4 (1.6)	-4.1^†^	0.226
ALT, U/L	38.9 (4.6)	33.9 (3.8)	-5.0∗	32.6 (3.3)	24.4 (2.4)	-8.2^†^	0.204
GGT, U/L	49.2 (4.0)	46.4 (4.6)	-2.8	49.6 (5.3)	27.6 (3.5)	-22.0^†^	E_T_ < W_T_^†^
Surrogate markers of NASH and fibrosis							
FAST-score	0.195 (0.026)	0.136 (0.022)	-0.060^†^	0.188 (0.023)	0.076 (0.017)	-0.112^†^	E_T_ < W_T_∗
FIB-4 index	0.986 (0.058)	0.979 (0.051)	-0.007	1.082 (0.071)	1.180 (0.087)	+0.097∗	E_T_ > W_T_∗
NF-score	-2.283 (0.144)	−2.429 (0.139)	-0.146	-2.021(0.200)	-1.833 (0.227)	+0.188	E_T_ > W_T_∗
Insulin resistance and lipid profile							
FPG, mg/dl	98.8 (1.8)	97.3 (1.7)	-1.5	105.9 (4.8)	97.5 (3.5)	-8.3∗	E_T_ < W_T_∗
HOMA-IR	2.96 (0.25)	2.64 (0.24)	-0.32∗	2.95 (0.40)	1.73 (0.28)	-1.22^†^	E_T_ < W_T_∗
TG, mg/dl	154.5 (13.5)	134.0 (10.4)	-20.5∗	144.1 (13.1)	90.5 (10.3)	-53.6^†^	E_T_ < W_T_∗
NEFA^ǂ^, Eq/L	0.62 (0.04)	0.5 (0.03)	-0.1^†^	0.50 (0.04)	0.59 (0.04)	+0.09	E_T_ > W_T_^†^
Daily dietary intake							
TEI^ǂ^, kcal/day	2,032.8 (64.7)	2,064.9 (62.0)	+32.1	2,282.8 (98.0)	1,599.4 (37.9)	-683.4^†^	E_T_ < W_T_^†^
Carbo, g/day	263.7 (8.8)	272.6 (7.7)	+8.9	279.8 (17.8)	209.3 (5.6)	-70.5^†^	E_T_< W_T_^†^
Protein^†^, g/day	72.6 (3.0)	69.5 (1.8)	-3.2	84.0 (3.3)	71.8 (2.7)	-12.2^†^	0.878
Fat, g/day	62.7 (2.6)	60.5 (2.3)	-2.3	68.9 (4.5)	46.2 (2.1)	-22.7^†^	E_T_ < W_T_^†^

Means (SEMs). Significant difference: ∗*p* <0.05; ^†^*p* <0.01. Within-group changes over time, between baseline and 3 months, for all variables were compared using paired *t* tests. Independent *t* tests or ANCOVA^ǂ^, with adjustment for baseline, were used to compare the changes between the groups. ALT, alanine aminotransferase; AST, aspartate transaminase; Carbo, carbohydrate; FAST-Score, FibroScan-AST Score; FPG, fasting plasma glucose; GGT, gamma-glutamyl transpeptidase; HOMA-IR, homeostasis model assessment-insulin resistance; NAFLD, non-alcoholic fatty liver disease; NEFA, non-esterified fatty acids; NF-Score, NAFLD fibrosis score; TEI, total energy intake; TG, triglyceride; WC, waist circumference.

**Table 2:** Outcomes of subgroups in 45 obese men with NAFLD who participated in an exercise regimen (E_sub_) or weight-loss regimen (W_sub_).

	E_sub_ (E_S_)			W_sub_ (W_S_)			ΔEs *vs*. ΔWs
Parameter	Before	After	Δ	Before	After	Δ	*p*
n	24			21			
Age, years	49.7 (1.5)			53.2 (2.2)			0.174
Attendance, %	85.8 (1.4)			86.5 (1.9)			0.750
Anthropometric characteristics							
Body weight, kg	83.3 (1.8)	81.6 (1.9)	-1.7^†^	83.3 (2.3)	74.3 (2.1)	-9.0^†^	ES < WS^†^
BMI, kg/m^2^	28.1 (0.5)	27.5 (0.5)	-0.6^†^	29.5 (0.5)	26.3 (0.5)	-3.2^†^	ES < WS^†^
WC, cm	97.7 (1.4)	94.3 (1.5)	-3.3^†^	100.0 (1.8)	90.1 (1.7)	-9.9^†^	ES < WS^†^
Fat mass, kg	21.3 (1.0)	19.9 (1.0)	-1.4^†^	21.1 (1.1)	16.1 (1.0)	-5.0^†^	ES < WS^†^
Lean mass, kg	62.0 (1.2)	61.6 (1.2)	-0.3	62.3 (1.7)	58.2 (1.6)	-4.1^†^	ES > WS^†^
Liver enzyme activities							
AST, U/L	23.6 (1.4)	22.1 (1.0)	-1.5	24.8 (1.2)	19.8 (1.3)	-5.0^†^	0.058
ALT, U/L	29.3 (2.7)	25.6 (1.8)	-3.7∗	30.0 (2.3)	23.6 (2.3)	-6.3∗	0.379
GGT, U/L	49.7 (6.5)	46.4 (6.2)	-3.3∗	42.3 (4.1)	23.5 (2.2)	-18.8^†^	ES < WS^†^
Surrogate markers of NASH and fibrosis							
FAST-score	0.145 (0.025)	0.087 (0.014)	-0.058^†^	0.173 (0.020)	0.055 (0.012)	-0.118^†^	ES < WS∗
FIB-4 index	0.900 (0.056)	0.885 (0.044)	-0.015	1.107 (0.090)	1.175 (0.111)	+0.068	0.188
NF-score	-2.353 (0.205)	-2.564 (0.176)	-0.211	-2.128 (0.237)	-1.965 (0.226)	+0.163	ES > WS∗
Insulin resistance and lipid profile							
FPG, mg/dl	99.8 (3.0)	96.0 (2.7)	-3.8^†^	102.1 (4.5)	94.8 (1.8)	-7.4∗	0.378
HOMA-IR	2.81 (0.36)	2.26 (0.31)	-0.55^†^	2.96 (0.43)	1.53 (0.18)	-1.43^†^	ES < WS^†^
TG, mg/dl	2.06 (0.05)	2.00 (0.05)	-0.06∗	2.13 (0.04)	1.92 (0.04)	-0.21^†^	ES < WS^†^
NEFA^ǂ^, Eq/L	0.59 (0.04)	0.50 (0.04)	-0.09^†^	0.47 (0.04)	0.59 (0.04)	+0.13∗	ES > WS^†^
Daily dietary intake							
TEI^†^, kcal/day	2,194.3 (81.1)	2,172.3 (93.2)	-21.9	2,378.8 (115.8)	1,588.5 (49.4)	-790.2^†^	ES > WS^†^
Carbo, g/day	274.0 (9.2)	273.0 (10.7)	-1.0	297.6 (22.5)	205.3 (6.1)	-92.3^†^	ES > WS^†^
Protein^ǂ^, g/day	79.7 (4.7)	72.9 (3.0)	-6.8	85.6 (4.0)	72.0 (3.3)	-13.6^†^	0.247
Fat, g/day	68.2 (3.5)	65.1 (3.8)	-3.1	73.1 (5.4)	49.3 (2.6)	-23.8^†^	ES < WS^†^

Means (SEMs). Significant differences: ∗*p* <0.05; ^†^*p* <0.01. Within-group changes over time, between baseline and 3 months, for all variables were compared using paired *t* tests. Independent *t* tests or ANCOVAǂ, with adjustments for baseline, were used to compare the changes between the groups. ALT, alanine aminotransferase; AST, aspartate transaminase; Carbo, carbohydrate; FAST-Score, FibroScan-AST Score; FPG, fasting plasma glucose; γ-GT, gamma-glutamyl transpeptidase; HOMA-IR, homeostasis model assessment-insulin resistance; NAFLD, non-alcoholic fatty liver disease; NEFA, non-esterified fatty acids; NF-Score, NAFLD fibrosis score; TEI, total energy intake; TG, triglyceride; WC, waist circumference.

### Dietary intake

As shown in Table 1, Table 2, the intakes of total energy, carbohydrate, protein, and fat were significantly lower in the W groups but not in the E groups during the interventions.

The magnitudes of the changes in the TEI, carbohydrate intake, and fat intake were greater in the W_total_ and W_sub_ groups than in the E_total_ and E_sub_ groups, respectively.

### Anthropometric characteristics

There were significant decreases in BMI, WC, and fat mass in the E_total_ group, but body and lean mass did not change during the intervention. In the W_total_ group, the body weight, BMI, WC, fat mass, and lean mass decreased.

Comparisons of the magnitudes of the changes showed that lean mass was preserved in the E_total_ group, but decreased to a greater extent in the W_total_ group. The decreases in the other parameters were larger in the W_total_ group than in the E_total_ group (Table 1).

The changes in the anthropometric characteristics of the E_sub_ and W_sub_ groups (Table 2) were similar to those in the E_total_ and W_total_ groups. However, in the E_sub_ group, body weight significantly decreased during the intervention.

### Liver enzyme activities

As shown in Table 1, Table 2, the AST and ALT activities in the E_total_ group, the ALT and GGT activities in the E_sub_ group, and all 3 activities in the W_total_ and W_sub_ groups decreased during the interventions.

The magnitudes of the decreases in GGT activity were greater in the W_total_ and W_sub_ groups than in the E_total_ and E_sub_ groups, respectively.

### Surrogate markers of NASH and fibrosis

As shown in Table 1, Table 2, the FAST-Score decreased in all the groups during the interventions. However, although the W_total_ group showed an increase in FIB-4 index, the other 3 groups (E_total_, E_sub_, and W_sub_ groups) did not. In addition, there were no significant changes in NF-Score in any of the groups.

The decrease in the FAST-Score was larger in the W_total_ and W_sub_ groups than in the E_total_ and E_sub_ groups, respectively. For the FIB-4 index, there was a significant difference between the E_total_ and W_total_ groups, but not between the E_sub_ and W_sub_ groups. The NF-Score significantly differed between the groups.

**Fig. 2:**
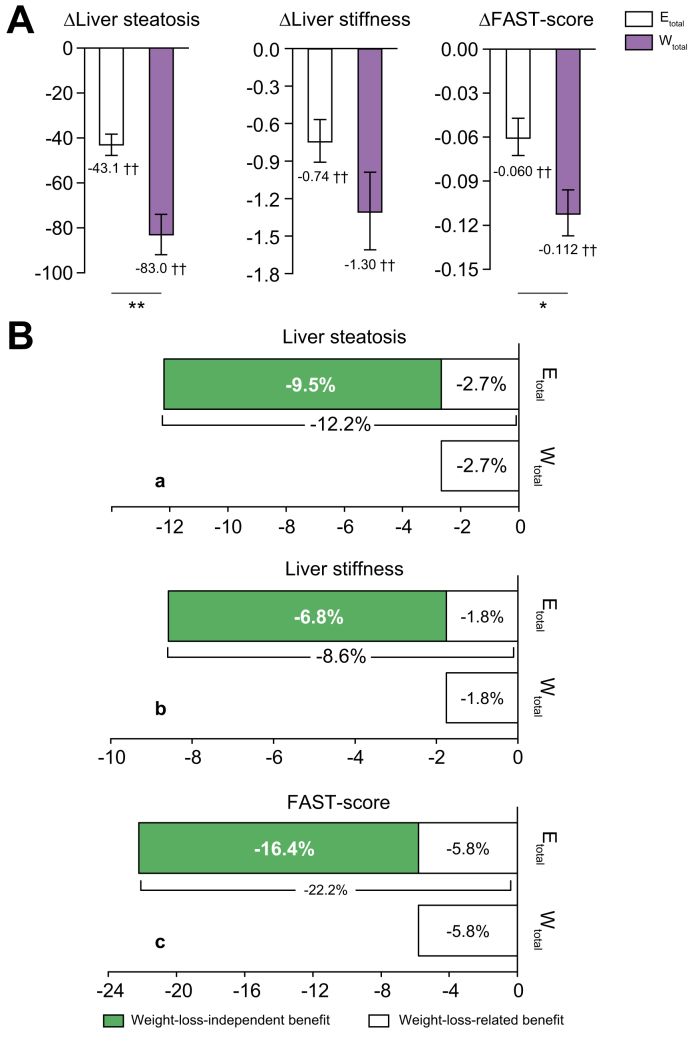
Comparison of weight-loss-independent benefits in terms of hepatic steatosis, stiffness, and FAST-Score. (A) Liver steatosis, stiffness, and FAST-Score. E_total_ n = 54, W_total_ n = 29. Dark grey bars: E_total_; light grey bars: W_total_ (means ± SEMs). ∗*p* <0.05, ∗∗*p* <0.01, between the groups. ^†^*p* < 0.05, ^‡^*p* <0.01, for baseline *vs.* 3 months. Within-group changes over time, between baseline and 3 months, for all variables were compared using paired *t* tests and independent *t* tests. (B) Weight-loss-independent benefits of exercise. E_total_ n = 54, W_total_ n = 29. The per 1% weight loss from each group, reduced percentages of liver steatosis, stiffness, and FAST-Score are shown. The exercise regimen reduced liver steatosis by 12.2%, liver stiffness by 8.6% and FAST-Score by 22.2% per 1% weight loss. In comparison, the weight-loss regimen reduced liver steatosis by 2.7%, liver stiffness by 1.8%, and FAST-Score by 5.8% per 1% weight loss. These results revealed that the exercise regimen reduced liver steatosis by an additional 9.5%, liver stiffness by an additional 6.8%, and FAST-Score by an additional 16.4%, when compared with those of the weight-loss-related benefit as a result of the weight-loss regimen (indicated by black bars). These additional benefits of the exercise regimen show independent effects of exercise that are not associated with weight loss.

The reduction in the level (%) of FAST-Score per 1% weight loss is shown in Fig. 2B. The exercise regimen was associated with a 16.4% weight-loss-independent effect as part of a total reduction in FAST-Score of 22.2%.

### Biochemical markers of NASH and fibrosis

**Fig. 3:**
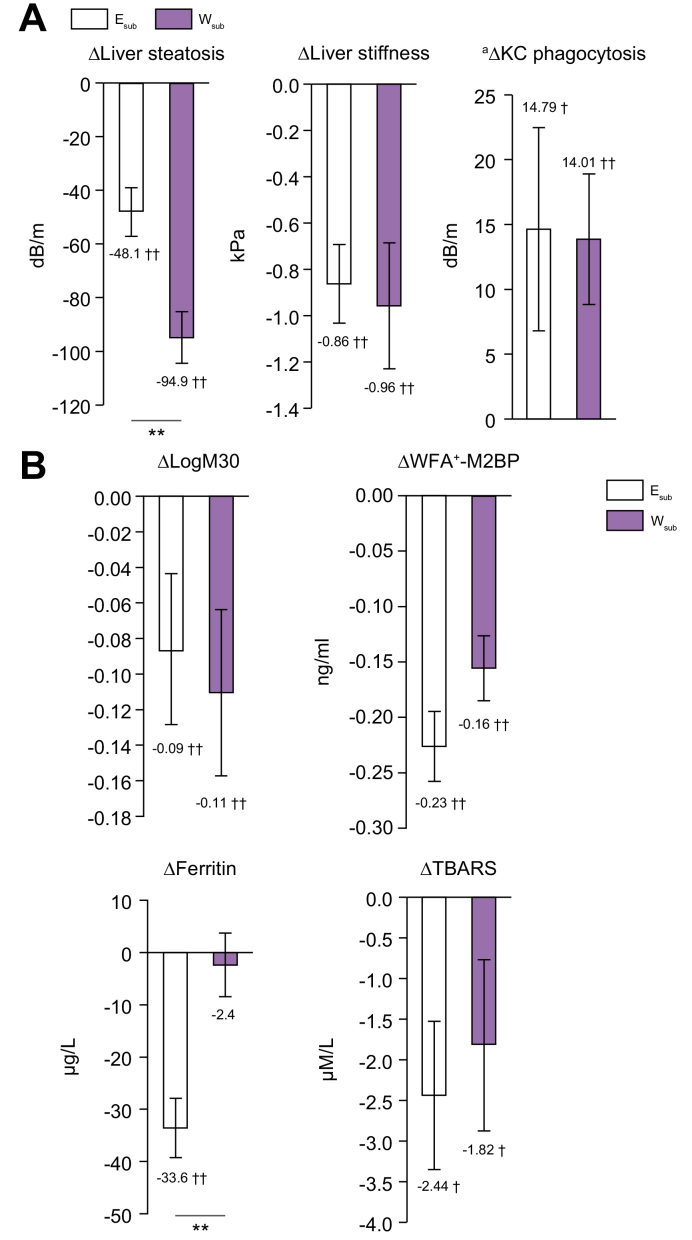
Changes in the hepatic pathophysiological conditions. (A) Liver steatosis, stiffness, and Kupffer cells (KC) phagocytosis. (B) Biochemical markers of NASH and fibrosis. E_sub_ n = 24, W_sub_ n = 21. Dark grey bars: E_sub_, light grey bars: W_sub_ (means ± SEMs). ∗*p* <0.05, ∗∗*p* <0.01, ^†^*p* <0.05, ^‡^*p* <0.01, for baseline *vs.* 3 months. Within-group changes over time, between baseline and 3 months, for all variables were compared using paired *t* tests and independent *t* tests, or ^a^ANCOVA. TBARS, thiobarbituric acid-reactive substances; WFA^+^-M2BP, *Wisteria floribunda* agglutinin-positive human Mac-2 binding protein.

All these biochemical markers (_log_M30: -3.9%, WFA^+^-M2BP: -62.4%, ferritin: -22.3%, and TBARS: -12.5%) decreased significantly in the E_sub_ group during the intervention. In addition, _log_M30 (-4.8%), WFA^+^-M2BP (-37.7%), and TBARS (-10.5%), but not ferritin, significantly decreased in the W_sub_ group (Fig. 3B).

The magnitude of the decrease in ferritin concentration was greater in the E_sub_ group than in the W_sub_ group (Fig. 3B).

### Insulin resistance and lipid profile

The HOMA-IR, TG, and NEFA in the E_total_ group, and the FPG, HOMA-IR, and TG in the W_total_ group decreased during the interventions (Table 1). Furthermore, all 4 parameters significantly changed in both the E_sub_ and W_sub_ groups (Table 2).

When the magnitudes of the changes were compared, the changes in NEFA concentration were larger in the E_total_ and E_sub_ groups, whereas the decreases in the other parameters were larger in the W_total_ group (Table 1). However, the magnitude of the decreases in FPG did not significantly differ between the E_sub_ and W_sub_ groups (Table 2).

### Liver stiffness, steatosis, and KC phagocytosis

Significant decreases in liver steatosis and stiffness were identified in the E_total_ group of -15.0% and -12.9%, in the W_total_ group of -28.1% and -20.2% (Fig. 2A), in the E_sub_ group of -17.0% and -15.4%, and in the W_sub_ group of -31.1% and -17.5% (Fig. 3A) during the interventions. KC phagocytosis significantly increased in the E_sub_ group by 13.2%, and in the W_sub_ group by 9.0% (Fig. 3A).

The magnitudes of decreases in liver steatosis were greater in the W_total_ and W_sub_ groups than in the E_total_ and E_sub_ groups, respectively, but the changes in liver stiffness and KC phagocytosis did not differ.

The reduction in the levels (%) of liver fat and stiffness per 1% weight loss is shown in Fig. 2B. The exercise regimen had a 9.5% weight-loss-independent effect to induce liver fat loss, of a total of 12.2%, and a 6.8% weight-loss-independent effect to induce a reduction in liver stiffness, of a total of 8.6%.

### Organokines

**Fig. 4:**
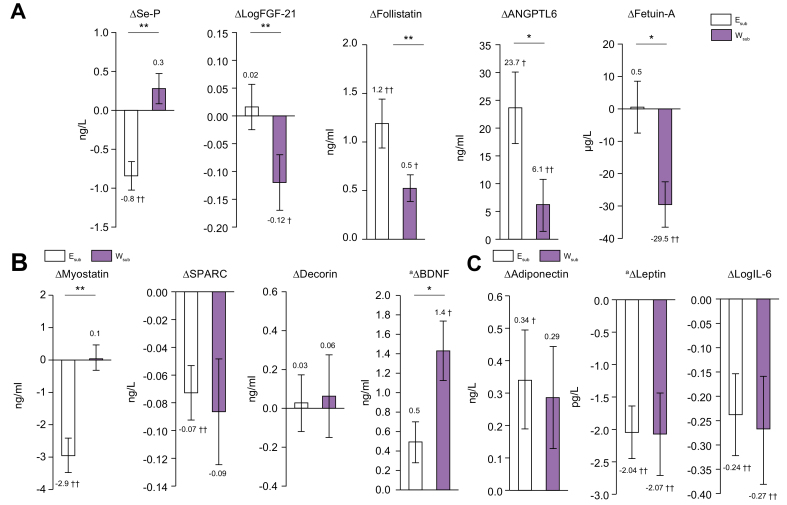
Changes in the organokine levels. (A) Hepatokines. (B) Myokines. (C) Adipokines. E_sub_ n = 24 and W_sub_ n = 21. Dark grey bars: E_sub_; light grey bars: W_sub_ (means ± SEMs). ∗*p* <0.05, ∗∗*p* <0.01, ^†^*p* <05, ^‡^*p* <0.01 for baseline *vs.* 3 months. Within-group changes over time, between baseline and 3 months, for all variables were compared using paired *t* tests and independent *t* tests, or ^a^ANCOVA. ANGPTL6, angiopoietin-like Protein 6; BDNF, brain-derived neurotrophic factor; FGF-21, fibroblast growth factor-21; Se-P, selenoprotein-P; SPARC, secreted protein acidic and rich in cysteine.

As shown in Fig. 4, the hepatokines Se-P (-10.6%), follistatin (+17.1%), and ANGPTL6 (+36.1%) significantly changed in the E_sub_ group, as did the myokines myostatin (-21.1%) and SPARC (-6.3%), and the adipokines adiponectin (+8.9%), leptin (-25.5%), and _log_IL-6 (-12.4%) during the intervention. In the W_sub_ group, there were significant changes in the hepatokines _log_FGF-21 (-5.2%), follistatin (-7.0%), ANGPTL6 (+9.0%), and fetuin-A (-10.5%); and the adipokines leptin (-35.0%) and _log_IL-6 (-13.9%); but there were no significant changes in the myokines.

The magnitude of the changes in the hepatokines Se-P, _log_FGF-21, follistatin, ANGPTL6, and fetuin-A and that in the myokines myostatin and BDNF significantly differed between the E_sub_ and W_sub_ groups, but that in the adipokines did not.

### Nrf2 target gene expression

**Fig. 5:**
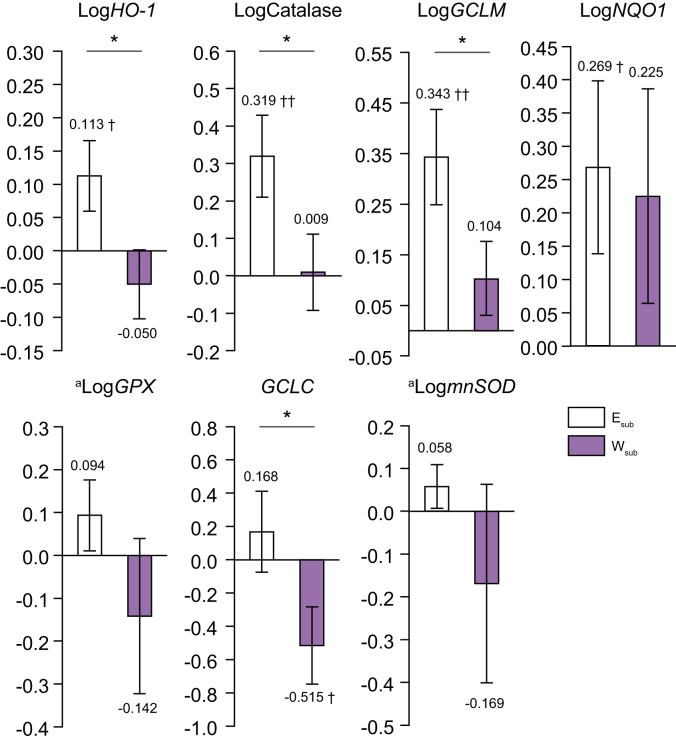
Expression levels of Nrf2 target genes. E_sub_ n = 24, W_sub_ n = 21. Dark grey bars: E_sub_; light grey bars: W_sub_ (means ± SEMs). ∗*p* <0.05, ∗∗*p* <0.01, ^†^*p* <0.05, ^‡^*p* <0.01, for baseline *vs.* 3 months. Within-group changes over time, between baseline and 3 months, for all variables were compared using paired *t* tests and independent *t* tests, or ^a^ANCOVA. *GCLC*, glutamate-cysteine ligase catalytic subunit; *GCLM*, glutamate-cysteine ligase modifier subunit; *GP*_*X*_*2*, glutathione peroxidase 2; *HO1*, heme oxygenase; *mnSOD*, manganese superoxide; *NQO1*, NADH quinone oxidoreductase; Nrf2, nuclear factor erythroid 2-related factor 2.

Figure 5 shows the changes in the PBMCs expression levels of 7 Nrf2 target genes (heme oxygenase-1, *HO1*; catalase; glutamate-cysteine ligase modifier subunit, *GCLM*; NAD (P) H-quinone oxidoreductase, *NQO1*; glutathione peroxidase, *GPx*; glutamate-cysteine ligase catalytic subunit, *GCLC*; and manganese superoxide dismutase, *mnSOD*) in the E_sub_ and W_sub_ groups during the interventions.

The expression of these 4 genes (_log_*HO1*: +7.5%, _log_catalase: +18.5%, _log_*GCLM*: +51.0%, and _log_*NQO1*: +22.4%) increased in the E_sub_ group, but that of *GCLC* (-10.3%) decreased in the W_sub_ group.

The magnitudes of the changes in expression of 4 genes (_log_*HO1*, _log_catalase, _log_*GCLM*, and *GCLC*) were greater in the E_sub_ group than in the W_sub_ group.

### Muscle strength

The results on muscle strength are described in Figure S1.

## Discussion

The principal findings of the present study can be summarised as follows. (1) In comparison with the weight-loss regimen, the exercise regimen maintained muscle mass better, while inducing only modest reductions in body and fat mass, and it also altered the circulating concentrations of a number of organokines (Fig. 4). (2) Ultrasound elastography revealed that the exercise regimen reduced liver steatosis by an additional 9.5%, liver stiffness by an additional 6.8%, and FAST-Score by an additional 16.4% *vs.* the weight-loss regimen (Fig. 2B). (3) The exercise regimen appeared to have induced anti-inflammatory and anti-oxidative stress responses, through activation of Nrf2 (Fig. 5).

In the present study, a weight loss of only 1.2% was induced by the 3-month exercise regimen (Table 1), but there were significant improvements in a number of NAFLD risk factors, including reductions in liver steatosis and stiffness (Fig. 2A). Our study group has been working on intervention studies related to exercise and/or a diet regimen for about 30 years. Through this experience, we are confident of the various psychological and physical benefits of exercise, but we found that if exercise is performed with the sole purpose of weight loss, the effect is very slight. Of course, the effect depends on the exercise duration, intensity, and modality, but the mean weight loss through our program consisting of walking and light jogging for 3 months always shows similar results of slight weight loss.^17^ We aimed to determine the mechanisms whereby this exercise regimen prevents the progression of hepatic steatosis and fibrosis, independent of weight loss.

Sarcopenia, which involves the loss of muscle mass and strength, is a risk factor for the development and progression of NAFLD, which implies the existence of a muscle–liver metabolic axis.^12^^,^^18^ In this study, almost no change in muscle mass occurred (+0.1 kg) during the exercise regimen, whereas a 3.9 kg reduction in muscle mass occurred during the weight-loss regimen (Table 1). In addition, muscle strength was increased by 11.6% during the former regimen, whereas it decreased by 12.1% during the latter regimen (Fig. S1). A loss of muscle mass or fat accumulation is associated with a loss of insulin sensitivity, facilitating the progression of liver steatosis.^19^ Furthermore, the hyperinsulinaemia induced by insulin resistance activates connective tissue growth factors in hepatic stellate cells, thereby contributing to the progression of liver fibrosis.^19^ Losses of skeletal muscle mass and function are frequently identified and are closely associated with liver pathology in NAFLD patients.^18^ In addition, the loss of muscle mass accompanying NAFLD increases the risks of NASH^18^ and death from liver-related diseases, such as cirrhosis and liver cancer,^20^ which is also consistent with the existence of a muscle–liver axis.

Skeletal muscle secretes myokines, which mediate crosstalk between the liver and adipose tissue.^21^ The participants in the E_sub_ group showed reductions in the myostatin and SPARC concentrations (Fig. 4B). Myostatin is an inhibitor of muscle growth^21^ and a promoter of liver fibrosis.^22^ Furthermore, the circulating myostatin is high in patients with both cirrhosis and sarcopenia.^22^ High myostatin concentration is associated with insulin resistance, which is in turn associated with muscular atrophy, resulting in poor exercise capacity and metabolic defects.^21^^,^^22^ Myostatin concentration has been shown to negatively correlate with lean mass in healthy adults,^23^ high levels can reflect an age-related reduction in muscle mass,^24^ the concentration is reduced by aerobic exercise,^21^ and myostatin is involved in the onset of insulin resistance as a result of lack of exercise.^21^ The circulating SPARC concentration is high in NAFLD patients who are likely to progress to the advanced stages of liver fibrosis, and an exercise-induced reduction may lead to a significant improvement in NAFLD, through the suppression of necrosis and inflammation in the liver.^25^

Hepatokines are liver-derived secreted factors that have been linked to NAFLD and insulin resistance. We found that the Se-P decreased and those of ANGPTL6 and follistatin increased in the E_sub_ group (Fig. 4A). Se-P is an inhibitor of insulin signalling in the liver and skeletal muscle.^26^ Fetuin-A, a hepatokine, also contributes to the onset and exacerbation of NAFLD,^27^ and the circulating Se-P and fetuin-A concentrations are high in patients with NAFLD.^26^^,^^27^ In this study, both interventions ameliorated insulin resistance (Table 2), but the Se-P was reduced by exercise, whereas the fetuin-A was reduced by the weight-loss regimen. These changes suggest that the regimens may ameliorate insulin resistance by distinct mechanisms. ANGPTL6 increases insulin sensitivity and leanness as a result of an increase in energy expenditure, and also contributes to the prevention of fat accumulation in the liver.^28^^,^^29^ Follistatin is known to be an inhibitor of myostatin, and is linked to muscle hypertrophy and to muscle maintenance under conditions of energy deprivation. Furthermore, it may also help prevent the progression of liver steatosis in NAFLD by its effects on skeletal muscle.^28^^,^^30^

The adipokine profiles of the participants were improved by both regimens (Fig. 4C). Interestingly, although the change in fat mass was lower during the exercise regimen than during the weight-loss regimen, the former was associated with a significant increase in circulating adiponectin, and reductions in IL-6 and leptin that were similar to those induced by the latter regimen. Therefore, we propose that exercise might induce a functional recovery in adipose tissue. The amelioration of this adipokine imbalance might also ameliorate insulin resistance in adipose tissue, which would in turn reduce lipotoxicity and lead to a reduction in systemic NEFA^31^ (Table 2).

In obese patients with NAFLD, metabolic endotoxaemia may occur because of the lower capacity of KC to phagocytose foreign material.^12^ In addition, NAFLD may be accompanied by metabolic endotoxaemia, which is caused by dysbiosis and greater production of the bacterial component lipopolysaccharide (LPS).^32^ Bacterially derived molecules, including LPS, have been reported to cause impairments in the composition and function of the liver and skeletal muscle via the gut–liver and gut–muscle axes, respectively.^32^^,^^33^ In the presence of hyperleptinemia, LPS is recognised as a foreign material, and stimulates nuclear factor-κB signalling and induces inflammation.^34^ This may exacerbate liver fibrosis and sarcopenia. KC phagocytosis is important for the defence against the effects of intestinal bacterially derived LPS, which promote hepatic steatosis and fibrosis.^32^ In this study, improvement in KC phagocytosis (Fig. 3A) may be important for the effect of exercise to prevent the progression of NAFLD, independent of weight loss.

Another important finding is that exercise activates the transcription factor Nrf2, which regulates detoxification and antioxidant defence in the liver, and hence protects against noxious stimuli.^35^ The E_sub_ group showed considerable increases in the expression of the Nrf2 target genes *HO1*, catalase, and *GCLM* (Fig. 5). In a previous study, we demonstrated that Nrf2 inhibits the development of NASH by inhibiting steatosis, inflammatory cell infiltration, and fibrosis in the liver at an early stage.^35^ In addition, Nrf2 inhibits the activation of the innate immune system by LPS^36^; therefore, a reduction in Nrf2 activity may increase LPS-induced inflammatory liver injury, alongside a reduction in KC phagocytosis.^36^ However, exercise has been shown to activate Nrf2 to an extent that is proportional to its intensity.^37^ Improvements in liver inflammation and fibrosis induced by the activation of liver Nrf2 may be implicated in the effect of the exercise to ameliorate liver defects in patients with NAFLD. In addition, Nrf2 can contribute to improvements in lipid profile, glucose tolerance, fat accumulation, and insulin sensitivity in the liver through activation of the liver kinase B1-AMP-activated protein kinase pathway and downstream genes.^38^

Our previous study^9^ showed the beneficial effects of increasing moderate-to-vigorous-intensity physical activity (MVPA) for the management of NAFLD. Consistent with these results, the present study has shown that higher intensity and volume of exercise are effective means of managing NAFLD. Participants in the E_large_ group, who more than doubled their amount of MVPA over a 3-month period, showed more marked amelioration of liver steatosis, reductions in liver enzyme activities, and improvements in organokines compared with those in the E_small_ group (Table S1). In addition, we have demonstrated that an increase in MVPA is an effective means of ameliorating both fat accumulation and fibrosis in the liver (Table S5).

There are limitations to be overcome in this study. First, with regard to the collected data from each intervention it should be noted that this was not designed as a randomised controlled trial, and this is likely to lead to spurious causality and bias. Also, our interventions were designed for a single-sex population. Only male participants were involved, and therefore it is difficult to make broader generalisations based on these results.

In conclusion, an exercise regimen maintained muscle mass and modified the circulating concentrations of many biologically active organokines. In addition, it increased phagocytosis by KC and activated Nrf2. The exercise prevented liver steatosis and fibrosis in NAFLD, independent of weight loss. Therefore, exercise, and especially MVPA, should be used to help prevent and treat NAFLD to improve prognosis.

## Financial support

This work was supported in part by Grants-in-Aid for Scientific Research from the 10.13039/501100001700Ministry of Education, Culture, Sports, Science and Technology, Japan (Grant Numbers 18K17918, 18H03172, 20H04119, and 20K11644). No sponsor had a role in the study design, data collection, analysis or interpretation of the data, or in the writing of the manuscript or the decision to submit the paper for publication.

## Authors’ contributions

Substantial contributions to study conception and design: S.O., J.S. Data acquisition: S.O., T.T., B.K., F.U., S.I., T.I. Data analysis: S.O., F.U., S.I. Data interpretation: S.O., T.S., T.I., H.S., J.S. Drafting of the article: S.O., J.S. Critical revision of the article for important intellectual content: K.T., T.S., H.S. All authors had full access to the study data and had final responsibility for the decision to submit for publication.

## Conflict of interest

The authors declare no conflicts of interest that pertain to this work.

Please refer to the accompanying ICMJE disclosure forms for further details.
