# Supplementary material for: Weight-loss-independent benefits of exercise on liver steatosis and stiffness in Japanese men with NAFLD
Source: JHEP Rep. 2021 Feb 10;3(3):100253. doi: 10.1016/j.jhepr.2021.100253 (PMC8059085; doi:10.1016/j.jhepr.2021.100253)
Supplement: Multimedia component 1 [file mmc1.pdf]

Weight loss-independent benefits of exercise on liver steatosis and stiffness in Japanese men with NAFLD

Sechang Oh, Takehiko Tsujimoto, Bokun Kim, Fumihiko Uchida, Hideo Suzuki, Seiichiro Iizumi, Tomonori Isobe, Takeji Sakae, Kiyoji Tanaka, Junichi Shoda

Table of contents

Supplementary methods....2

Supplementary results....6

Supplementary figure legend....11

Fig. S1....12

Table S1....13

Table S2....15

Table S3....16

Table S4....17

Table S5....18

Supplementary methods

Table S1

Group classification

The E_{sub} group was divided into two subgroups: a group that undertook a large amount of exercise (E_{large}, n = 12) and a group that undertook a small amount of exercise (E_{small}, n = 12), so that we could determine whether the amount of moderate-to-vigorous-intensity physical activity (MVPA) performed had a bearing on the benefits of exercise. This would permit us to suggest the optimal amount of exercise that would be required to achieve clinical improvement.

Attendance rate

There were 12 dietary restriction classes for participants in the weight-loss groups and 36 exercise classes for participants in the exercise groups. We calculated the number of classes each participant attended as a percentage.

Analysis of physical activity

To evaluate the daily amount and intensity of exercise, participants wore a single-axis accelerometer (Lifecorder; Suzuken Co Ltd, Nagoya, Japan) during their periods of activity from 2 weeks prior to the start of the intervention until the end of the intervention period. We used the MVPA volume to divide subjects (n=24) as "large" (n=12) and "small" (n=12) amounts of exercise.

Statistics

Statistical analysis was conducted using SPSS ver. 25.0 (IBM Corp, Armonk, NY, USA). The results are presented as means (SEMs) for the raw data. To characterize intra-group changes over time, dependent variables were analyzed using Wilcoxon's rank sum test. Differences between groups were identified using the Mann-Whitney U-test. Finally, when data were adjusted for baseline values, ranked analyses of covariance (ANCOVA) was conducted. All tests were two-sided and statistical significance was accepted at $P < 0.05$.

Table S5

Statistics

Statistical analysis was conducted using SPSS ver. 25.0 (IBM Corp). A multivariate linear regression model was constructed using stepwise selection that considered all the variables that were significant in univariate analyses. Statistical significance was accepted at $P < 0.05$.

Fig. S1

Isometric leg muscle strength

Maximal strength of the isometric contraction of knee extension was measured using a Biodex III dynamometer (Biodex Medical Systems Inc., Shirley, NY, USA). The participants performed a warm-up with professional trainers before the test, and then were seated and tightly secured using chest, pelvic, and thigh straps, with their backs supported at an angle of 120°. The knee was extended at an angle of 60° for the isometric assessment. The peak torque (Nm) of three maximal extension efforts was determined as the maximal strength. Each isometric contraction was held for 3 seconds, with intervening 15-second pauses. The results were reported as absolute values (Nm) and normalized body weight (Nm/kg). These methods have been previously described in detail (Kim, B. et al. Changes in muscle strength after diet-induced weight reduction in adult men with obesity: a prospective study. Diabetes Metab Syndr Obes 10, 187–194 2017)

Supplementary results

Table S1

Baseline analysis

The age, attendance rate, and anthropometric characteristics of the groups did not significantly differ. However, there were significant differences in the AST activity and FAST-Score between the E_{large} and E_{small} groups. These data were analyzed with adjustment for baseline values.

Dietary intake

The intakes of total energy, carbohydrate, protein, and fat did not significantly change in either the E_{large} or E_{small} groups during the intervention. The magnitudes of these changes did not differ between the E_{large} and E_{small} groups.

Physical activity

The amount of MVPA and the number of steps taken increased significantly in both the E_{large} and E_{small} groups during the intervention, but the amount of low-intensity physical activity did not change. The magnitudes of the increases in the MVPA and the number of steps taken were greater in the E_{large} group than in the E_{small} group.

Anthropometric characteristics

The lean mass did not change in either the E_{large} or E_{small} groups, but other parameters significantly decreased in the E_{large} group, and WC and fat mass decreased in the E_{small} group during the intervention. The magnitudes of the changes in these parameters did not significantly differ between the groups.

***Liver enzyme activities*:**

The activities of all three enzymes (AST, ALT, and γ-GT) in the E_{large} group significantly decreased during the intervention. However, there were no significant changes in the E_{small} group. Comparisons of the E_{large} and E_{small} groups revealed that the magnitude of the change in γ-GT activity was greater in the E_{large} group than in the E_{small} group.

Surrogate markers of NASH and fibrosis

The FAST-Score decreased in the E_{large} group, but not in the E_{small} group during the intervention. There were no significant changes in FIB-4 index or NF-Score during the intervention period in any of the groups.

Comparisons between the E_{large} and E_{small} groups revealed that the magnitude of the decrease in the FAST-Score was greater in the E_{large} group than in the E_{small} group. However, for the FIB-4 index and NF-Score, there were no significant differences.

Biochemical markers of NASH and fibrosis

TBARS did not significantly change in either the E_{large} or E_{small} groups, but other parameters (ferritin, M30, and WFA^{+}-M2BP) decreased in both the E_{large} and E_{small} groups during the intervention. The magnitudes of these decreases did not differ between the E_{large} and E_{small} groups.

Insulin resistance and lipid profile

In the E_{small} group, only FPG showed a significant decrease, and HOMA-IR and NEFA showed decreases in the E_{large} group. However, there were not significant differences in the changes in the E_{large} and E_{small} groups.

Liver stiffness, steatosis, and KC phagocytosis

The liver steatosis and stiffness of both the E_{large} and E_{small} groups significantly decreased during the intervention. KC phagocytosis decreased in the E_{large} group, but not in the E_{small} group.

Comparisons between the E_{large} and E_{small} groups revealed that the magnitude of the decrease in liver steatosis was greater in the E_{large} group than in the E_{small} group. However, there were no significant differences in the changes in the liver stiffness and KC phagocytosis between the groups.

Organokines

Of the 12 organokines assessed, five (Se-P, FGF-21, follistatin, myostatin, and leptin) in the E_{large} group and eight (Se-P, follistatin, ANGPTL6, myostatin, SPARC, leptin, adiponectin, and IL-6) in the E_{small} group significantly changed during the intervention.

Comparisons between the E_{large} and E_{small} groups revealed that the magnitudes of changes in the FGF-21, myostatin, and leptin concentrations were greater in the E_{large} group than in the E_{small} group.

Nrf2 target gene expression

In the E_{large} group, there were significant increases in catalase, *GCLM*, and *NQO1* expression levels; and in the E_{small} group, increases in catalase, *GCLM*, and *mnSOD* levels were found during the intervention. There were no significant differences in the magnitudes of the changes between the E_{large} and E_{small} groups.

Table S5

Multiple regression analysis was performed using the reduction in liver steatosis, as the dependent variable, to identify the pathophysiological factors associated with exercise. Only the increase in MVPA was found to independently influence the reduction in liver steatosis. In addition, multiple regression analysis was performed using the reduction in liver stiffness, which reflects the reduction in liver fibrosis and/or inflammation, as the dependent variable, to identify the pathophysiological factors associated with exercise. Only the reduction in liver steatosis was found to independently affect the reduction in liver stiffness. These findings demonstrate that the increase in MVPA achieved using the exercise regimen was an effective means of reducing both fat storage and stiffness in the liver.

Fig. S1

Absolute values (Nm) and body weight-normalized (Nm/kg) isometric leg muscle strength were significantly increased in the E_{sub} group during the intervention, but not in the W_{sub} group.

Comparisons between the E_{sub} and W_{sub} groups revealed that the absolute values (Nm) and normalized body weight (Nm/kg) isometric leg muscle strength significantly differed.

Supplementary figure legends

Fig. S1. Changes in isometric leg muscle strength between baseline and the 3-month end-point in patients with NAFLD who participated in an exercise regimen (E_{sub} = 24) or a weight-loss regimen (W_{sub} = 21)

The dark grey bars represent E_{sub} and the light gray bars represent W_{sub} (means ± SEMs). ††*P* < 0.01, †*P* < 0.05: significant differences between baseline and month 3; ***P* < 0.01, **P* < 0.05: significant differences between the groups.

Fig. S1

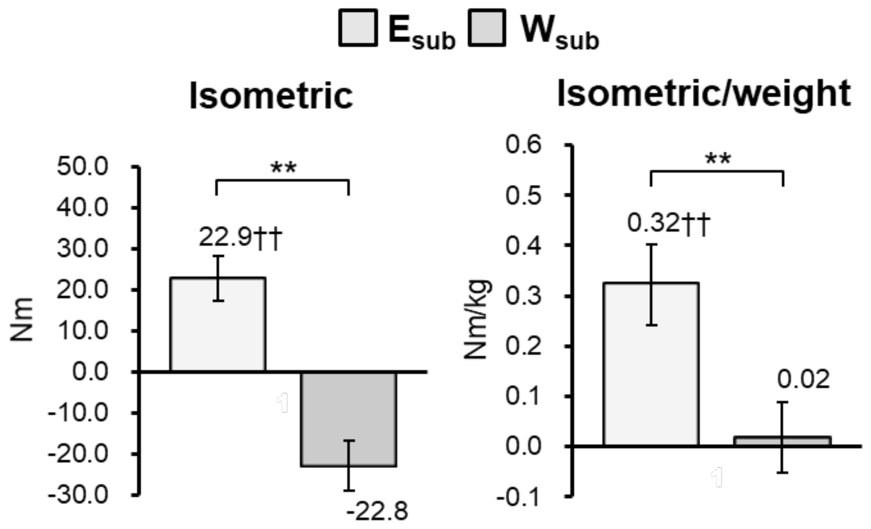

E$_{sub}$ W$_{sub}$
Isometric
Isometric/weight
50.0
40.0
30.0
20.0
10.0
0.0
-10.0
-20.0
-30.0
Nm
**
22.9††
-22.8
0.6
0.5
0.4
0.3
0.2
0.1
0.0
-0.1
Nm/kg
**
0.32††
0.02

Table S1. Outcomes of subgroups in 24 obese men with NAFLD who participated in an exercise regimen and undertook either a large amount or (E_{large}) or a small amount (E_{small}) of moderate-to-vigorous physical activity

	E_{large} [E_L]			E_{small} [E_S]			E_L *vs.* E_S
Parameter	Baseline	After	Change	Baseline	After	Change	*P*
n		12			12		
Age, years		50.7 (2.0)			48.7 (2.2)		0.504
Attendance, %		85.4 (2.2)			86.1 (2.0)		0.817
**Physical Activity**							
MVPA, min·d^{-1}	44.1 (4.6)	89.7 (6.4)	+45.6**	49.1 (4.6)	59.8 (6.4)	+10.8**	$E_L > E_S$**
LPA, min·d^{-1}	231.9 (20.4)	242.9 (17.0)	+10.9	265.0 (14.6)	245.4 (10.1)	−19.6	0.068
Steps·d^{-1}	5,946.9 (6179)	11,070.5 (840)	+5,123.6**	7,418.0 (546)	8,954.0 (766)	+1,536.0*	$E_L > E_S$**
**Daily Dietary Intake**							
TEI, kcal·d^{-1}	2,135.0 (146.1)	2,144.4 (142.8)	+9.3	2,253.5 (74.4)	2,200.3 (125.8)	−53.2	0.713
Carbohydrate, g·d^{-1}	280.3 (13.9)	286.1 (15.2)	+5.8	267.7 (12.5)	260.0 (14.6)	−7.8	0.478
[a]Protein, g·d^{-1}	77.5 (8.9)	70.8 (4.4)	−6.7	82.0 (3.3)	75.0 (4.0)	−7.0	0.671
Fat, g·d^{-1}	61.6 (4.6)	51.5 (2.8)	−10.1	74.9 (4.8)	66.7 (4.5)	−8.1	0.977
**Anthropometric Characteristics**							
Body Weight, kg	83.5 (2.5)	81.3 (2.5)	−2.2**	83.1 (2.7)	81.8 (3.0)	−1.2	0.514
BMI, kg·m^{-2}	28.5 (0.7)	27.8 (0.7)	−0.8**	27.7 (0.7)	27.3 (0.8)	−0.4	0.478
WC, cm	98.6 (1.9)	94.8 (1.5)	−3.8**	96.8 (2.0)	93.9 (2.6)	−2.9**	0.319
Fat Mass, kg	21.7 (1.5)	20.0 (1.3)	−1.7**	20.9 (1.4)	19.9 (1.6)	−1.0*	1.000
Lean Mass, kg	61.8 (1.5)	61.3 (1.6)	−0.5	62.1 (1.8)	61.9 (1.8)	−0.2	0.410
**Hepatic Parameters**							
Liver Steatosis, dB·m^{-1}	297.0 (15.7)	230.1 (10.4)	−66.9**	266.9 (9.7)	237.7 (14.5)	−29.3**	$E_L > E_S$*
Liver Stiffness, kPa	5.76 (0.36)	4.84 (0.30)	−0.92**	5.39 (0.29)	4.56 (0.21)	−0.83**	0.663
KC phagocytosis, dB·m^{-1}	135.7 (8.7)	149.3 (9.8)	+13.6*	128.5 (14.2)	150.2 (6.3)	+21.7	0.730
**Liver Enzyme Activities**							
[a]AST, U·L^{-1}	26.9 (2.2)	23.2 (1.0)	−3.7*	20.3 (1.4)	21.0 (1.6)	+0.7	$E_L > E_S$*
ALT, U·L^{-1}	33.7 (4.4)	26.9 (2.7)	−6.8*	24.8 (2.8)	24.3 (2.5)	−0.6	$E_L > E_S$*
γ-GT, U·L^{-1}	53.3 (10.5)	46.5 (8.4)	−6.8*	46.3 (10.7)	46.6 (8.4)	+0.3	$E_L > E_S$*
**Biochemical markers of NASH and Fibrosis**							
Ferritin, μg·L^{-1}	133.1 (22.2)	101.5 (18.1)	−31.6**	168.6 (26.7)	133.0 (20.9)	−35.6**	0.590
TBARS, μM·L^{-1}	20.7 (1.6)	18.0 (1.3)	−2.7	18.5 (1.3)	16.3 (0.6)	−2.1	0.379
M30, U·L^{-1}	283.5 (70.6)	200.2 (39.8)	−83.3**	204.8 (24.8)	177.5 (23.7)	−27.3*	0.932
WFA^{+}-M2BP, ng·mL^{-1}	0.40 (0.05)	0.17 (0.03)	−0.2**	0.35 (0.05)	0.12 (0.03)	−0.2**	0.755
**Surrogate Markers of NASH and Fibrosis**							
FAST-Score	0.202 (0.039)	0.093 (0.016)	−0.109**	0.089 (0.022)	0.081 (0.025)	−0.007	$E_L > E_S$*
FIB-4 index	0.915 (0.06)	0.880 (0.037)	−0.035	0.885 (0.098)	0.891 (0.082)	+0.006	0.465
NF-Score	−2.413 (0.261)	−2.630 (0.180)	−0.217	−2.293 (0.326)	−2.498 (0.310)	−0.206	0.973
**Adipokines**							
Leptin, pg·L^{-1}	8.57 (1.06)	5.72 (0.80)	−2.85*	7.38 (0.98)	6.21 (0.96)	−1.17*	$E_L > E_S$*
Adiponectin, ng·L^{-1}	3.58 (0.35)	3.83 (0.51)	+0.25	4.10 (0.62)	4.53 (0.73)	+0.43*	0.378
IL-6, pg·mL^{-1}	1.43 (0.23)	1.32 (0.34)	−0.11	2.49 (0.82)	0.81 (0.20)	−1.68*	0.061
**Hepatokines**							
Se-P, ng·L^{-1}	7.06 (0.65)	6.18 (0.66)	−0.88**	8.03 (0.83)	7.23 (0.78)	−0.81*	0.928
FGF-21, pg·mL^{-1}	229.8 (53.3)	282.2 (64.0)	+52.4*	327.4 (55.5)	253.4 (41.0)	−74.0	$E_L > E_S$**
Follistatin, ng·mL^{-1}	7.1 (0.2)	8.5 (0.5)	+1.4**	7.0 (0.2)	7.9 (0.4)	+0.9**	0.519
ANGPTL6, ng·mL^{-1}	64.2 (4.7)	82.9 (9.8)	+18.7	67.5 (4.1)	96.1 (11.4)	+28.4**	0.332
Fetuin-A, ng·L^{-1}	288.9 (18.4)	274.5 (15.6)	−14.4	264.9 (12.4)	278.9 (14.9)	+14.1	0.132
**Myokines**							
Myostatin, pg·mL^{-1}	14.7 (2.2)	10.4 (1.7)	−4.3**	12.8 (1.7)	11.2 (1.9)	−1.6*	$E_L > E_S$**
SPARC, ng·L^{-1}	1.14 (0.16)	1.08 (0.15)	−0.06	1.06 (0.15)	0.97 (0.13)	−0.09*	0.429
Decorin, ng·mL^{-1}	3.76 (0.36)	3.87 (0.23)	0.11	3.92 (0.20)	3.86 (0.24)	−0.06	0.577
BDNF, ng·mL^{-1}	2.7 (0.1)	3.3 (0.3)	+0.6	2.7 (0.2)	3.1 (0.3)	+0.4	0.699
**Insulin Resistance and Lipid Profile**							
FPG, mg·dL^{-1}	96.8 (3.1)	94.3 (2.0)	−2.5	102.8 (5.0)	97.8 (5.1)	−5.0*	0.242
HOMA-IR	3.4 (0.6)	2.7 (0.5)	−0.7*	2.2 (0.3)	1.8 (0.3)	−0.5	0.799
TG, mg·dL^{-1}	156.9 (26.6)	150.2 (32.8)	−6.8	112.4 (20.7)	91.0 (10.2)	−21.4	0.799
[a]NEFA, Eq·L^{-1}	0.58 (0.06)	0.46 (0.06)	−0.1*	0.61 (0.06)	0.55 (0.05)	−0.1	0.242
**Nrf2 Target Gene Expression**							
Catalase	0.659 (0.09)	2.121 (0.57)	+1.462*	0.5309 (0.07)	1.357 (0.34)	+0.826*	0.331
GPX2	0.034 (0.01)	0.038 (0.01)	+0.005	0.021 (0.010)	0.036 (0.010)	+0.015	0.552
HO1	0.509 (0.2)	0.572 (0.2)	+0.062	0.325 (0.050)	0.482 (0.100)	+0.156	0.566
GCLM	0.082 (0.02)	0.137 (0.04)	+0.055*	0.077 (0.04)	0.138 (0.03)	+0.060*	0.487
mnSOD	83.8 (10.4)	97.3 (23.5)	+13.5	71.6 (6.5)	101.6 (14.0)	+30.0*	0.190
NQO1	1.068 (0.88)	4.981 (3.51)	+3.912*	0.332 (0.2)	0.515 (0.21)	+0.182	0.074

GCLC	4.869 (0.644)	5.26 (0.54)	+0.395	5.048 (0.476)	5.012 (0.454)	−0.036	0.905

Means (SEMs). Significant differences: *P < 0.05; **P < 0.01. Within-group changes over time, between baseline and 3 months, for all dependent variables were analyzed using Wilcoxon's rank sum test. Mann-Whitney U tests or [a]ranked analyses of covariance (ANCOVA), with adjustments for baseline data, were used to compare the changes between groups. **LPA**, light physical activity; **TEI**, total energy intake; **BMI**, body mass index; **WC**, waist circumference; **AST**, aspartate transaminase; **ALT**, alanine aminotransferase; **γ-GT**, gamma-glutamyl transpeptidase; **TBARS**, thiobarbituric acid-reactive substances; **WFA$^+$-M2BP**, *Wisteria floribunda* agglutinin-positive human Mac-2-binding protein; **FAST-Score**, FibroScan-AST Score; **NF-Score**, NAFLD fibrosis score; **IL-6**, interleukin 6; **Se-P**, selenoprotein-P; **FGF-21**, fibroblast growth factor 21; **ANGPTL6**, angiopoietin-like 6; **SPARC**, secreted protein acidic and rich in cysteine; **BDNF**, brain-derived neurotrophic factor; **FPG**, fasting plasma glucose; **HOMA-IR**, homeostatic model assessment-insulin resistance; **TG**, triglyceride; **NEFA**, non-esterified fatty acids; ***GPX2***, glutathione peroxidase 2; ***HO1***, heme oxygenase-1; ***GCLM***, glutamate-cysteine ligase modifier subunit; ***mnSOD***, manganese superoxide dismutase; ***NQO1***, NAD (P) H: quinone oxidoreductase; ***GCLC***, glutamate-cysteine ligase catalytic subunit.

Table S2. Laboratory methods used in the study

Name	Method
AST	Japan Society of Clinical Chemistry transferable method
ALT	Japan Society of Clinical Chemistry transferable method
γ-GT	Japan Society of Clinical Chemistry transferable method
FPI	Chemiluminescent immunoassay method
Ferritin	Chemiluminescent immunoassay method
FPG	Enzymatic colorimetric method
TG	Enzymatic method
NEFA	Enzymatic method

AST, aspartate aminotransferase; **ALT**, alanine aminotransferase; **γ-GT**, gamma-glutamyl transpeptidase; **FPI**, fasting plasma insulin**; FPG**, fasting plasma glucose; **TG**, triglyceride; **NEFA**, non-esterified fatty acids.

Table S3. Commercial enzyme-linked immunosorbent assay and electrochemiluminescence kits used in the study

Name	Manufacturer
TBARS	Cayman Chemical, Ann Arbor, USA
IL-6	R&D Systems, Minneapolis, USA
Leptin	R&D Systems, Minneapolis, USA
FGF-21	R&D Systems, Minneapolis, USA
Fetuin-A	R&D Systems, Minneapolis, USA
SPARC	R&D Systems, Minneapolis, USA
BDNF	R&D Systems, Minneapolis, USA
Follistatin	R&D Systems, Minneapolis, USA
M30	Peviva AB, Bromma, Sweden
Adiponectin	Sekisui Medical, Tokyo, Japan
Se-P	Cusabio Biotech, Wuhan China
Myostatin	Cusabio Biotech, Wuhan China
ANFPTL6	Biovision, Milpitas, USA
Decorin	Aviscera Bioscience, Santa Clara, USA
WFA^+-M2BP	Immuno-Biological Lab, Kunma, Japan
Fetuin-A	BioVender Lab Med, Brno, Czech Republic

TBARS, thiobarbituric acid-reactive substances; **IL-6**, interleukin 6; **FGF-21,** fibroblast growth factor 21; **SPARC**, secreted protein acidic and rich in cysteine; **BDNF**, brain-derived neurotrophic factor; **Se-P,** selenoprotein-P; **ANGPTL6**, angiopoietin-like 6; **WFA^+-M2BP**, *Wisteria floribunda* agglutinin-positive human Mac-2 binding protein.

Table S4. Primers used for quantitative real-time PCR

Gene name	Forward	Reverse
GAPDH	5′AGGTGAAGGTCGGAGTCA3′	5′GGTCATTGATGGCAACAA3′
HO1	5′CCAGGCAGAGAATGCTGAGT3′	5′GTAGACAGGGGCGAAGACTG3′
Catalase	5′ACCAGGGCATCAAAACCTTT3′	5′CCGGATGCCATAGTCAGGAT3′
GCLM	5′GAAGAAGATATTTTTCCTGTCATTGAT3′	5′CCATTCATGTATTGAAGAGTGAATTT3′
NQO1	5′CTGATCGTACTGGCTCACTC3′	5′AACAGACTCGGCAGGATAC3′
GPx2	5′ACAACCACCCGGGACTTCA3′	5′CCAAATTGGTTGCAAGGGAA3′
GCLC	5′ATTCCTGACATTCAAGCGCAC3′	5′TTCCTCTACTTTTCACAATGACCGA3′
mnSOD	5′GGGTTGGCTTGGTTTCAATA3′	5′CTGATTTGGACAAGCAGCAA3′

GAPDH, glyceraldehyde 3-phosphate dehydrogenase; ***HO1***, heme oxygenase-1; ***GCLM***, glutamate-cysteine ligase modifier subunit; ***NQO1***, NAD(P)H: quinone oxidoreductase; ***GPX2***, glutathione peroxidase 2; ***GCLC***, glutamate-cysteine ligase catalytic subunit; ***mnSOD***, manganese superoxide dismutase
