# Supplementary material for: Weight-loss-independent benefits of exercise on liver steatosis and stiffness in Japanese men with NAFLD
Source: JHEP Rep. 2021 Feb 10;3(3):100253. doi: 10.1016/j.jhepr.2021.100253 (PMC8059085; doi:10.1016/j.jhepr.2021.100253)
Supplement: Multimedia component 2 [file mmc2.pdf]

JHEP|Reports

JHEP Reports

CTAT methods

Tables for a “Complete, Transparent, Accurate and Timely account” (CTAT) are now mandatory for all revised submissions. The aim is to enhance the reproducibility of methods.

- Only include the parts relevant to your study
- Refer to the CTAT in the main text as ‘Supplementary CTAT Table’
- Do not add subheadings
- Add as many rows as needed to include all information
- Only include one item per row

If the CTAT form is not relevant to your study, please outline the reasons why:

1.1 Antibodies

Name	Citation	Supplier	Cat no.	Clone no.

1.2 Cell lines

Name	Citation	Supplier	Cat no.	Passage no.	Authentication test method

1.3 Organisms

Name	Citation	Supplier	Strain	Sex	Age	Overall n number

1.4 Sequence based reagents

Name	Sequence	Supplier
HO-1 Forward	5′-CCAGGCAGAGAATGCTGAGT-3′	Sigma-Aldrich
HO-1 Reverse	5′-GTAGACAGGGGCGAAGACTG-3′	Sigma-Aldrich
Catalase Forward	5′- ACCAGGGCATCAAAACCTTT-3′	Sigma-Aldrich
Catalase Reverse	5′-CCGGATGCCATAGTCAGGAT-3′	Sigma-Aldrich
GCLM Forward	5′-GAAGAAGATATTTTTCCTGTCATTGAT-3′	Sigma-Aldrich
GCLM Reverse	5′-CCATTCATGTATTGAAGAGTGAATTT-3′	Sigma-Aldrich
NQO1 Forward	5′ -CTGATCGTACTGGCTCACTC-3′	Sigma-Aldrich
NQO1 Reverse	5′-AACAGACTCGGCAGGATAC-3′	Sigma-Aldrich
GPx Forward	5′-ACAACCACCCGGGACTTCA-3′	Sigma-Aldrich
GPx Reverse	5′-CCAAATTGGTTGCAAGGGAA-3′	Sigma-Aldrich
GCLC Forward	5′-ATTCCTGACATTCAAGCGCAC-3′	Sigma-Aldrich
GCLC Reverse	5′-TTCCTCTACTTTTCACAATGACCGA-3′	Sigma-Aldrich
mnSOD Forward	5′-GGGTTGGCTTGGTTTCAATA-3′	Sigma-Aldrich
mnSOD Reverse	5′-CTGATTTGGACAAGCAGCAA-3′	Sigma-Aldrich
GAPDH Forward	5′-AGGTGAAGGTCGGAGTCA-3′	Sigma-Aldrich
GAPDH Reverse	5′-GGTCATTGATGGCAACAA-3'	Sigma-Aldrich

1.5 Biological samples

Description	Source	Identifier

1.6 Deposited data

Name of repository	Identifier	Link

1.7 Software

Software name	Manufacturer	Version
SPSS	IBM, Armonk, NY, USA	25.0
Excel Eiyo-Kun	Kenpakusya, Tokyo, Japan	4.0

1.8 Other (*e.g.* drugs, proteins, vectors etc.)

1.9 Please provide the details of the corresponding methods author for the manuscript:

Sechang Oh, Ph.D.
Faculty of Medicine, University of Tsukuba, 1-1-1 Tennodai, Tsukuba, Ibaraki 305-8575, Japan
Phone: (+81) 29 853 3291; Fax: (+81) 29 853 3291
E-mail: ohsechang@md.tsukuba.ac.jp

2.0 Please confirm for randomised controlled trials all versions of the clinical protocol are included in the submission. These will be published online as supplementary information.

N/A
